# Randomized controlled trials in central vascular access devices: A scoping review

**DOI:** 10.1371/journal.pone.0174164

**Published:** 2017-03-21

**Authors:** Mari Takashima, Gillian Ray-Barruel, Amanda Ullman, Samantha Keogh, Claire M. Rickard

**Affiliations:** 1 Alliance for Vascular Access Teaching and Research (AVATAR) group, Menzies Health Institute Queensland, Griffith University, Nathan, Queensland, Australia; 2 School of Nursing & Institute of Health and Biomedical Innovation (IHBI), Queensland University of Technology, Brisbane, Australia; University of Liverpool, UNITED KINGDOM

## Abstract

**Background:**

Randomized controlled trials evaluate the effectiveness of interventions for central venous access devices, however, high complication rates remain. Scoping reviews map the available evidence and demonstrate evidence deficiencies to focus ongoing research priorities.

**Method:**

A scoping review (January 2006–December 2015) of randomized controlled trials evaluating the effectiveness of interventions to improve central venous access device outcomes; including peripherally inserted central catheters, non-tunneled, tunneled and totally implanted venous access catheters. MeSH terms were used to undertake a systematic search with data extracted by two independent researchers, using a standardized data extraction form.

**Results:**

In total, 178 trials were included (78 non-tunneled [44%]; 40 peripherally inserted central catheters [22%]; 20 totally implanted [11%]; 12 tunneled [6%]; 6 non-specified [3%]; and 22 combined device trials [12%]). There were 119 trials (68%) involving adult participants only, with 18 (9%) pediatric and 20 (11%) neonatal trials. Insertion-related themes existed in 38% of trials (67 RCTs), 35 RCTs (20%) related to post-insertion patency, with fewer trials on infection prevention (15 RCTs, 8%), education (14RCTs, 8%), and dressing and securement (12 RCTs, 7%). There were 46 different study outcomes reported, with the most common being infection outcomes (161 outcomes; 37%), with divergent definitions used for catheter-related bloodstream and other infections.

**Conclusion:**

More high quality randomized trials across central venous access device management are necessary, especially in dressing and securement and patency. These can be encouraged by having more studies with multidisciplinary team involvement and consumer engagement. Additionally, there were extensive gaps within population sub-groups, particularly in tunneled devices, and in pediatrics and neonates. Finally, outcome definitions need to be unified for results to be meaningful and comparable across studies.

## Introduction

Central venous access devices (CVADs) provide access to the greater vascular system to administer therapy contraindicated to be given peripherally, for longer term treatment, and for venous monitoring and blood sampling [1]. Patients requiring CVADs are heterogeneous, with varying ages, acute and chronic illnesses, across hospitals and in community care. There are many different CVAD types inserted for different treatment requirements; for example, long or short term duration, and continuous or intermittent therapy. CVAD care is complex and multi-faceted; clinicians from diverse clinical specialties are involved in their insertion and management [1–4].

Despite the prevalence of CVADs in acute and chronic care, serious insertion and management complications associated with CVADs continue to be prevalent [5–10]. Many complications, including bloodstream infection, are considered a preventable source of patient harm and have a significant negative impact on patients and healthcare costs [11]. Developing, testing and implementing effective interventions to prevent CVAD-associated harm are important considerations for healthcare researchers, clinicians, and patients. In order to improve the quality and safety of CVADs, many randomized controlled trials (RCTs) have been conducted to evaluate the effectiveness of healthcare interventions.

The RCT is considered to be the “gold standard” for evaluating the effectiveness of an intervention, as it provides reliable evidence with minimal risk of bias compared to other study designs [12]. Clinicians refer to results of RCTs and systematic reviews to guide clinical decision-making, however, this may be challenged if results cannot be generalized to their population because of too few RCTs, small sample sizes, poor reporting, and a lack of clear effect [13]. Therefore, it is important for researchers to develop their research agenda based upon the identified priority areas and clinical needs, while minimizing unnecessary duplication of research and associated costs.

Scoping reviews are used to map the existing research in a given field and to highlight the gaps in evidence [14–17]. They examine the breadth of literature published, with the aim of providing insights and guidance for clinicians and researchers on where to focus their research [17]. The aim of this scoping review was to reveal the RCTs conducted in relation to CVADs in the past decade and to synthesize the patient populations and clinical settings studied, interventional themes addressed, and the outcome measures used.

## Methods

### Review framework

This scoping review was conducted using the framework outlined by Arksey and O’Malley [14] and modified by the Cochrane Public Health Group [14, 16], which is used extensively [18–20]. The framework consists of the following steps: 1. Identify the research question; 2. Identify relevant studies; 3. Select studies for inclusion; 4. Sort, collate, and analyze data; and 5. Summarize and report results [14]. Consensus on methodology and inclusion/exclusion criteria were established during the first phase of the project to ensure consistency in decision making.

### Identify the research questions

The objectives of the review were to answer the following questions:

1. What RCTs have been conducted on the effectiveness of interventions to improve CVAD outcomes within contemporary literature (< 10 years), and what were the study characteristics?
2. What CVAD types, population demographics, and clinical settings were included?
3. What were the interventional themes studied?
4. What outcome measures were reported?

### Identify relevant studies

A systematic search was undertaken. Studies were eligible for inclusion if they met predefined inclusion criteria: (1) employed an RCT design; (2) evaluated interventions to improve outcomes associated with CVADs; and (3) were published between 1 January 2006 and 31 December 2015. CVADs were limited to peripherally inserted central catheter (PICC), non-tunneled CVAD (NTCVAD), tunneled CVAD, and totally implantable venous access device (TIVAD) used in any patient population. Umbilical catheters and dialysis catheters were excluded from the review as they are used in special population groups only. Studies comprising more than one CVAD type were included in the scoping review if the article specified the results for each device type, allowing the results of the target devices to be extracted. All participant ages and settings (inpatient and ambulatory) were included. We excluded non-RCT designs (including cross-over design), secondary analysis of RCTs, non-English studies without an English abstract, and non-human studies.

### Search strategy

The search strategy was developed with the assistance of a university health sciences librarian. Databases were systematically and independently searched on the 19^th^ February, 2016. Databases included PubMed (US National Library of Medicine), Cochrane Central Register of Controlled Trials (the Cochrane Library), Cumulative Index to Nursing and Allied Health Literature (CINAHL), and Medline (US National Library of Medicine). (See S1 File for search terms). Reference lists of retrieved systematic reviews were reviewed to ensure all potential RCTs were included.

### Study selection and data extraction

References were imported into EndNote^™^ (Clarivate Analytics) and then sorted and examined throughout the inclusion/exclusion process [14]. Each reference, title and abstract was initially dual-screened by a pair of two independent investigators (MT, GB, AU, SK). Two independent reviewers then reviewed the full text of selected studies for inclusion/exclusion criteria, and, if eligible, extracted the data using a standardized data extraction form. If the group was unclear of the inclusion, all four reviewers met until an agreement was reached.

### Data sorting, collating, and analysis

Data were extracted into a Microsoft Excel file to organize the data under the following headings: first author; year of publication; country; first author profession; title of journal; method of randomization; study population (inpatient/ambulant; neonates/pediatrics/adults; clinical specialities); sample size; discipline of CVAD inserter; interventional theme; outcome measures; key findings; and grant funding. The risk of bias of the individual studies was not formally assessed in the same way as a systematic review [21]. This is because of the differing goals of scoping reviews, which are designed to illuminate the breadth rather than the depth of available research [14, 15]. However, this review incorporates the appraisal of the methodological quality by including assessment of randomization methods and outcome definitions [22]. Authors were not contacted for further information or to obtain full-text.

### Summarizing and reporting results

**Fig 1:**
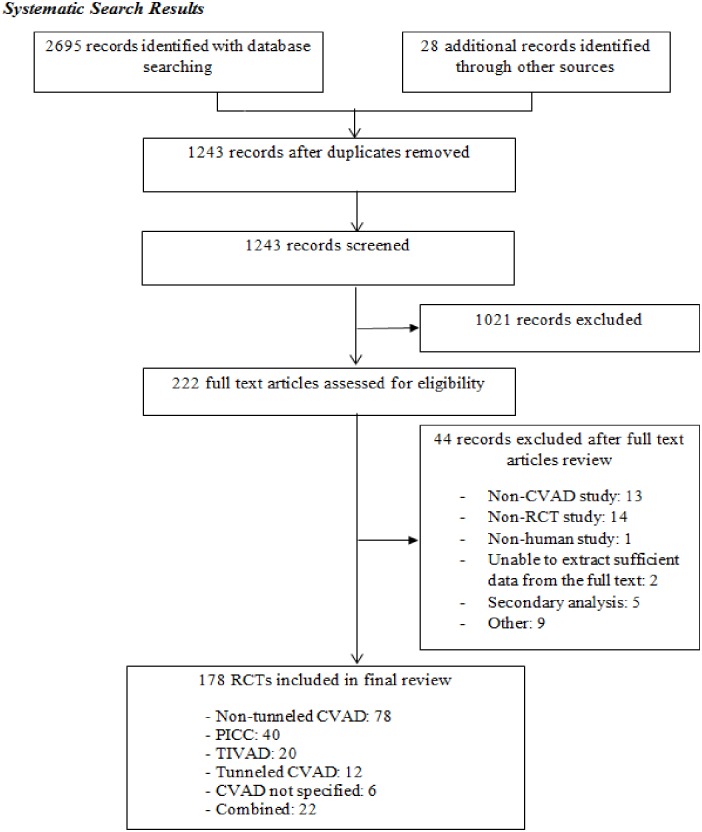
Flowchart of articles screened for inclusion in the scoping review. RCT: Randomized Controlled Trial; CVAD: Central Venous Access Device; PICC: Peripherally Inserted Central Catheter; TIVAD: Totally Implantable Vascular Access Device.

Preliminary tables were constructed to identify topics that had been the focus of many RCTs and topics that were lacking in evidence. Microsoft Excel was used to create graphs and tables. Fig 1 describes the flow of inclusion and exclusion for the study selection, in accordance with the referred Preferred Reporting Items for Systematic Reviews and Meta-Analyses (PRISMA) guidelines [23]. Database searching identified 2,695 RCTs and an additional 28 RCTs were added after reviewing the reference lists of 77 identified systematic reviews. After duplicates and studies with non-relevant abstracts were removed, 222 RCTs were reviewed. Twenty-nine RCTs provided abstract information only.

## Results

### Characteristics of included RCTs

The final review included 178 RCTs (78 non-tunneled [24–101]; 40 PICC [102–141]; 20 totally implanted [142–161]; 12 tunneled [162–173]; 6 non-specified [174–179]; 22 combined [180–201]; See S1 Table for included studies). The non-specified category was created to classify studies that did not specify the type of CVAD. There were nine pilot studies (6%) and 28 multi-center studies (16%). The largest number of RCTs were published in the USA (32 RCTs; 18%), followed by Italy (12 RCTs; 7%), and Brazil, Netherlands, France, and Germany (10 RCTs each; 6%).

First authors were mainly medical doctors (96 RCTs; 54%), followed by departmental information only (48 RCTs; 27%), and nurses (13 RCTs; 7%). Information regarding grant monies was listed as public/departmental source (43 RCTs; 24%), followed by missing/not stated (27 RCTs; 15%), or missing/abstract information only (25 RCTs; 14%). Industry grants accounted for 19 RCTs (11%), and 13 RCTs received both public and private funding (7%). Forty percent (n = 70) of RCTs used computer-generated allocation, which is the standard method of randomization [202]. However, 42 RCTs (24%) did not state the randomization method (excluding those that provided abstract information only), and some studies had questionable randomization methods, such as alternate allocation, random admission to ward allocation, and lottery method, which can potentially be vulnerable to subversion [202].

### Population demographics and clinical settings of included RCTs

Patient populations studied were mostly adults (119 RCTs: 68%) (See Table 1). In younger patients, neonates were the target population in 20 RCTs (11%) and pediatrics in 18 RCTs (10%). There was a difference in frequency of use of CVAD types in different populations. Non-tunneled CVADs were the most popular catheter type studied in adults (n = 23,435, 60% of all adult RCTs), compared to 24% of all pediatric RCTs (n = 431) and 4% of all neonate RCTs (n = 87). Most studied CVAD types for pediatrics and neonates were tunneled CVADs (n = 744, 41% of all pediatric RCTs) and PICCs respectively (n = 1572, 78% of neonate RCTs). Pediatrics comprised 4% (n = 1816) of the total sample size compared to adults (n = 38,979, 84%) and neonates (n = 2009, 4%).

**Table 1:** Population table of included RCTs (N = 178 studies, 46,258 participants).

	NTCVAD	PICC	TIVAD	Tunneled	Combined	CVAD-NS	Total
	n (%)	n (%)	n (%)	n (%)	n (%)	n (%)	n (%)
**Population (number of participants)**							
Adult	23,435 (60%)	2,742 (7%)	4,322 (11%)	917 (2%)	7,222 (19%)	341 (1%)	38,979 (100%)
Pediatrics	431 (24%)	240 (13%)	151 (8%)	744 (41%)	250 (14%)	0	1,816 (100%)
Neonates	87 (4%)	1,572 (78%)	0	0	350 (18%)	0	2,009 (100%)
Combined	240 (18%)	0	0	0	1089 (82%)	0	1,329 (100%)
Staff	724 (88%)	32 (4%)	0	0	0	65 (8%)	821 (100%)
Unknown	394 (30%)	250 (19%)	0	0	0	660 (51%)	1,304 (100%)
Total	25,311 (55%)	4,836 (10%)	4,473 (10%)	1,661 (4%)	8,911 (19%)	1,066 (2%)	46,258 (100%)
**Setting (number of studies)**							
Inpatient	65 (47%)	27 (19%)	11 (8%)	12 (9%)	20 (14%)	3 (2%)	138 (100%)
Outpatient	0	3 (38%)	4 (50%)	0	1 (13%)	0	8 (100%)
Both	0	2 (33%)	4 (67%)	0	0	0	6 (100%)
Staff	11 (85%)	1 (8%)	0	0	0	1 (8%)	13 (100%)
Not stated	2 (15%)	7 (54%)	1 (8%)	0	1 (8%)	2 (15%)	13 (100%)
Total	78 (44%)	40 (22%)	20 (11%)	12 (7%)	22 (12%)	6 (3%)	178 (100%)
Clinical setting (number of studies)							
Intensive Care Unit (ICU)	37 (61%)	19 (30%)	0	0	4 (7%)	1 (2%)	61 (100%)
Adult ICU	(35)	(2)	(0)	(0)	(1)	(1)	(39)
Pediatric ICU	(1)	(1)	(0)	(0)	(0)	(0)	(2)
Neonatal ICU	(1)	(16)	(0)	(0)	(3)	(0)	(20)
Hematology/Oncology	5 (10%)	4 (8%)	16 (33%)	10 (20%)	11 (22%)	3 (7%)	49 (100%)
Operating room	18 (90%)	0	0	0	1 (5%)	1 (5%)	20 (100%)
All patients requiring vascular access/outpatients/not stated	2 (10%)	10 (50%)	4 (20%)	1 (5%)	3 (15%)	0	20 (100%)
Education/Training facility	10 (91%)	0	0	0	0	1 (9%)	11 (100%)
Medical/Surgical	2 (25%)	3 (38%)	0	1 (12%)	2 (25%)	0	8 (100%)
Combined	4 (66%)	1 (17%)	0	0	1 (17%)	0	6 (100%)
Radiology department	0	3 (100%)	0	0	0	0	3 (100%)
Emergency department*	0	0	0	0	0	0	0
**Total**	78 (45%)	40 (22%)	20 (11%)	12 (7%)	22 (12%)	6 (3%)	178 (100%)

*Two studies were undertaken in ED in conjunction with other clinical areas, but no study was undertaken in ED alone.

RCT: Randomized Controlled Trial; CVAD: Central Venous Access Device; NTCVAD: Non-tunneled Central Venous Access Device; PICC: Peripherally Inserted Central Catheter; TIVAD: Totally Implantable Vascular Access Device; CVAD NS: Central Venous Access Device Not Specified

The most prevalent clinical specialties for CVAD RCTs were intensive care units (ICU) (61 RCTs; 34%), with a further 4 RCTs including some ICU patients, followed by hematology/oncology settings (49 RCTs; 27%). Most non-tunneled CVAD studies (78 RCTs) took place in ICUs (37 RCTs; 47%) or operating rooms (18 RCTs, 23%). PICCs were studied predominantly in ICUs (19 RCTs), of which the majority were neonatal ICU (16 RCTs; 84%). Hematology/oncology was the most prevalent clinical setting for TIVAD (16 out of 20 RCTs; 80%), tunneled CVAD (10 out of 12 RCTs; 83%), and combined catheter (11 out of 22 RCTs, 50%) studies. No studies were conducted solely in the emergency department (ED), but two RCTs included some ED devices. Staff education was the focus of 13 RCTs (7%); these were primarily simulation studies and only two detailed nursing education. The majority of CVADs were inserted by doctors or medical interns (75 RCTs; 42%). Vascular access nurses or vascular access teams including nurses inserted CVADs in 17 RCTs (9%), and radiologists inserted CVADS in 13 RCTs (7%).

### Interventional themes of included RCTs

**Fig 2:**
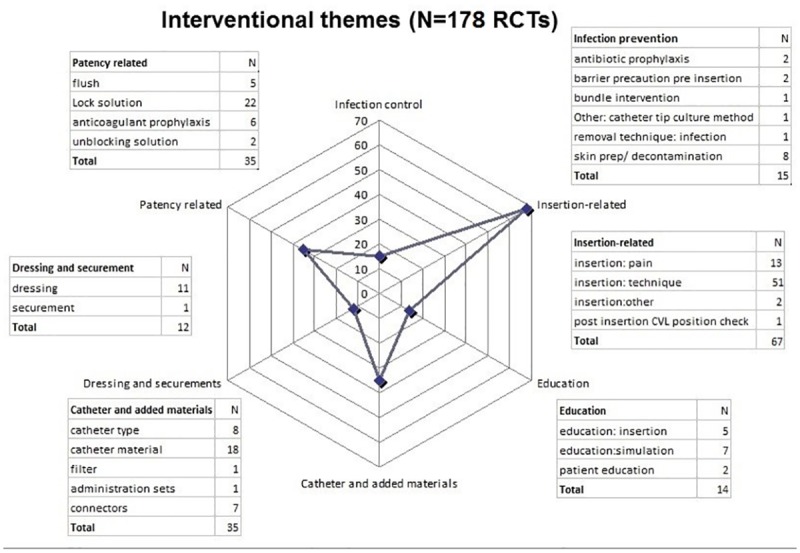
Interventional themes of included RCTs (N = 178 RCTs).

Among all CVAD types, insertion technique was the most common topic (51 RCTs, 29% of all studies), followed by lock solutions (22 RCTs, 12%) (See Fig 2, S2 and S3 Tables for detailed per CVAD type table). Only five studies examined flushing technique, in contrast to more common lock solution studies (22 RCTs). There were only one or two studies on each of the following topics: bundle interventions, securement, in-line filters, administration sets, unblocking solutions, and patient education.

There were 433 individual outcomes in this review, which were categorized into seven major themes (See Table 2). The most prevalent outcomes studied were: infective (n = 161, 37%), followed by catheter complications (n = 99, 23%), and catheter insertion outcomes (n = 101, 23%). These major categories were further subdivided into sub-categories (See S4 Table to see the variety of definitions of outcomes across the studies).

**Table 2:** Study outcomes (N = 433) of included RCTs (Detailed table per CVAD type in S4 Table).

Reported Study Outcomes		Totals
**Catheter complications, N (%)**		99 (22.9%)
Thrombosis	29 (6.7%)	
Occlusion	23 (5.3%)	
Mechanical failure ^a^	15 (3.4%)	
Early removal	14 (3.3%)	
Dwell time	12 (2.8%)	
Complication rate (not specified)	5 (1.2%)	
Local edema/ inflammation	1 (0.2%)	
**Patient outcomes, N (%)**		29 (6.6%)
Pain scores	14 (3.2%)	
Patient satisfaction/ Quality of life	6 (1.4%)	
Vital signs	3 (0.7%)	
Mortality rate	3 (0.7%)	
Psychological distress	1 (0.2%)	
Patient comprehension	1 (0.2%)	
Self-management ability	1 (0.2%)	
**Catheter insertion outcomes, N (%)**		101 (23.3%)
Successful placement measures	47 (10.9%)	
Insertion-related complications	39 (9.0%)	
Insertion success: performance scores	13 (3.0%)	
Use of ultrasound	1 (0.2%)	
Requirement for repositioning	1 (0.2%)	
**Infective outcomes, N (%)**		161 (37.2%)
Catheter-related blood stream infection	47 (10.9%)	
Catheter-related infection	29 (6.7%)	
CVAD tip colonization	29 (6.7%)	
Contamination/ colonization of non-catheter materials including skin and hub	20 (4.6%)	
Systemic infection/sepsis/ fever	18 (4.2%)	
Local infection/ exit-site infection/phlebitis	17 (3.9%)	
Microbial biofilm	1 (0.2%)	
**Patency-related outcomes, N (%)**		10 (2.3)
Patency	5 (1.2%)	
Thrombolytic/ fibrinolysis injection	4 (0.9%)	
Anticoagulant treatment	1 (0.2%)	
**Intervention-related outcomes, N (%)**		21 (4.8%)
Side effects/ tolerability	16 (3.7%)	
Bleeding	4 (0.9%)	
Skin necrosis	1 (0.2%)	
**Health service-related outcomes, N (%)**		12 (2.8%)
Health economics/cost	12 (2.8%)	
**TOTAL**		**433 (100%)**

^a^ Mechanical failures include migration, catheter defects, malfunction, infiltration, skin fixation failure, dislocation, fracture and other.

## Discussion

This scoping review is the first to systematically identify the gaps in the recent RCT evidence for CVAD use. Many studies included in the review (67 RCTs, 38%) focused on effective CVAD insertion practices, however, comparatively few evaluated maintenance strategies, such as infection prevention (15 RCTs, 8%) and dressing and securement (12 RCTs, 7%). Important areas such as securement, patient education, and bundled interventions lack RCT data.

The findings of this review accord with a recent scoping review of RCTs in peripheral vascular access catheters, which found catheter insertion strategies were extensively studied, but there was a lack of robust evidence to support post-insertion care and maintenance, including dressings and securement, flushing practices, and infection prevention strategies [203]. CVAD insertion complications occur in 0.4–4.5% of procedures, in comparison to post-insertion complications, which can occur in up to 25% of the device life [5, 204–206]. CVADs are designed for prolonged use compared to peripheral intravenous catheters, and more evidence for post-insertion care is needed to avoid unnecessary complications and the need for catheter replacement.

It is important that the same RCT questions and outcome variables are replicated in several RCTs in different clinical settings before a precise and reliable assessment of effect can be determined, preferably in a systematic review and meta-analysis. Our review highlighted that this depth of RCT testing is not yet present in the literature for all areas of CVAD management, with many single RCTs focusing on an intervention in one study only [25, 47, 58, 67, 70, 72, 91, 107, 108, 122, 156, 163, 168, 177, 181, 185, 187, 194, 195, 199]. The potential barriers to undertaking and publishing RCTs in vascular access are likely to be due to a lack of research knowledge, skills, and funding. However, many such issues could be resolved if vascular access teams and other health professionals affiliate with local academics and incorporate research and publishing within their service roles [207].

In addition, the scoping review revealed the variety of outcome definitions used, particularly for infection outcomes. For example, CRBSI as per the Centers for Disease Control and Prevention (CDC) definition; CRBSI defined by other references [208–214], catheter-related infection defined by the authors, catheter-related infection not specified, and catheter-related sepsis. Half of the CRBSI outcomes included within the scoping review were defined by references other than the CDC, with some references dating from the 1990s despite the included RCTs being published from 2006 onwards. Furthermore, many catheter-related infections were not defined. Such heterogeneous reporting of infective outcomes makes the comparison of studies problematic and, with the weak effect, cannot be extrapolated to clinical practice.

CVAD-associated thrombosis outcomes were also diagnosed differently across studies. In the review thrombosis was categorized into three categories: 1. Diagnosed and screened by instruments, such as venography, ultrasound, MRI or CT scans; 2. Diagnosed clinically and then scanned by instruments; and 3. Diagnosed clinically only. Around 33% of the thrombosis outcome studies did not screen patients with instruments. This could potentially lessen the effect when compared to the studies that screened for thrombosis for all patients. With variable definitions, it is difficult to relate the evidence to clinically important outcomes. There is a need for consensus on definition for catheter complications including mechanical and infective outcomes in order to have meaningful results that are comparable across studies.

Pediatrics and neonates have been understudied across all CVAD types. There were only 18 RCTs in pediatrics, 20 RCTs in neonates, and 3 in combined adults and pediatrics, compared to 119 adult RCTs. Previous systematic reviews have concluded that more evidence from this population group is required to reach significant results in meta-analysis [215, 216]. Neonatal and pediatric populations have different underlying anatomy and physiology and indications for CVAD insertion and use. It is not recommended to extrapolate results from adult RCTs to neonatal and pediatric populations, where physiological and technical reasons may modify the effect [217]. Therefore, pediatric and neonatal clinicians have inadequate evidence on which to base their vascular access practice, which could contribute to poorer outcomes for children, families and the healthcare system. More RCTs to provide evidence to support CVAD insertion and management decisions are urgently needed in this vulnerable population [217].

This scoping review has several limitations. Primarily, it did not examine the risk of bias as assessed in systematic reviews. However, the aim was to capture the current CVAD themes being studied, which this review successfully captured. Secondly, some references were analyzed from abstracts without full text available. This could have potentially misclassified some variables due to a lack of complete information. Thirdly, research abstracts published in a language other than English were excluded due to cost and time involved in translating material, so these results reflect only the English literature. However, this is the first scoping review published on RCTs on CVADs, and it has made a unique and significant contribution to the body of knowledge in this area. It provides a platform for prioritizing rigorous research in areas such as CVAD maintenance to provide high level evidence to inform guidelines and practice. Additionally, it has given future recommendations on methods and reporting of the randomization process and unification of outcome definitions for RCT findings to be significant and useful for systematic reviews.

## Conclusion

This scoping review has identified RCTs in a range of CVAD types to detect the gaps in evidence and highlight areas needing further research. Many RCTs focus on insertion-related themes, but there is a scarcity of RCTs in post-insertion care. There is also a need for consensus on outcome definitions to avoid heterogeneous outcomes, such as CRBSI, catheter-related infections, and thrombosis. Almost a quarter of RCTs did not state the randomization method, which may degrade the quality of the study. Researchers should report their results in accordance with the relevant CONSORT reporting guidelines including randomization method to ensure reliability [218].

More RCTs in post-insertion care are necessary, and these can be encouraged by having more interdisciplinary collaboration for CVAD research, including doctors, nurses, allied health professionals and patients. There are over 5 million CVADs inserted every year in the United States [219]. A recent systematic review of CVAD complications in pediatrics found that 25% of CVADs failed before completion of therapy [5]. The estimated proportion of catheter failure from all CVAD complications for adults is still unknown. In 2009, an estimated 18,000 central line-associated bloodstream infections occurred in ICU patients in the United States, with a single episode costing up to US$22,939 [220, 221]. These potentially preventable injuries cause direct harm to patients and place an enormous financial strain on healthcare institutions. This study has identified that more high quality evidence is necessary to inform CVAD maintenance practices to avoid such complications to reduce unnecessary CVAD replacements and associated cost.

## Supporting information

S1 File Search terms. (DOCX)

S1 Table Table of included studies. (DOCX)

S2 Table Table 3: Study themes by categories (N = 178 RCTs). (DOCX)

S3 Table Table 4: Study themes by CVAD type (N = 178 studies). *One catheter material study had also a theme of heparin flush. One removal technique study included insertion technique, but this is only included in one category. RCT: Randomized Controlled Trial; CVAD: Central Venous Access Device; NTCVAD: Non-tunneled Central Venous Access Device; PICC: Peripherally Inserted Central Catheter; TIVAD: Totally Implantable Vascular Access Device; CVAD NS: Central Venous Access Device Not Specified; PICU: Pediatric Intensive Care Unit; NICU: Neonatal Intensive Care Unit.
(DOCX)

S4 Table Table 5: Study outcomes by CVAD type (433 outcomes). RCT: Randomized Controlled Trial; CVAD: Central Venous Access Device; NTCVAD: Non-tunneled Central Venous Access Device; PICC: Peripherally Inserted Central Catheter; TIVAD: Totally Implantable Vascular Access Device; CVAD NS: Central Venous Access Device Not Specified; PICU: Pediatric Intensive Care Unit; NICU: Neonatal Intensive Care Unit; CDC: Centers of Disease Control; MRI: Magnetic Resonance Imaging; USS: Ultra sound sonography.
(DOCX)
