# Supplementary material for: Randomized controlled trials in central vascular access devices: A scoping review
Source: PLoS One. 2017 Mar 21;12(3):e0174164. doi: 10.1371/journal.pone.0174164 (PMC5360326; doi:10.1371/journal.pone.0174164)
Supplement: S1 File — (DOCX) [file pone.0174164.s001.docx]

**S1 File: Search terms**

((("central venous catheter"[Title/Abstract] OR "central venous access device"[Title/Abstract]) OR "central venous device"[Title/Abstract]) OR ("Vascular Access Devices"[Mesh] OR "Central Venous Catheters"[Mesh])) AND ("Randomized Controlled Trial"[Publication Type] OR "Randomized Controlled Trials as Topic"[Mesh]) AND (("2006/01/01"[PDAT] : "2015/12/31"[PDAT]) AND "humans"[MeSH Terms]) AND Randomized Controlled Trial[ptyp]

((("peripherally inserted central catheter"[Title/Abstract] OR "PICC"[Title/Abstract]) OR "Catheterization, Peripheral"[Mesh]) OR ("Vascular Access Devices"[Mesh] OR "Central Venous Catheters"[Mesh])) AND ("Randomized Controlled Trial"[Publication Type] OR "Randomized Controlled Trials as Topic"[Mesh]) AND (Randomized Controlled Trial[ptyp] AND ("2006/01/01"[PDAT] : "2015/12/31"[PDAT]) AND "humans"[MeSH Terms])

 ((((implant* venous[Title/Abstract]) AND port[Title/Abstract])) OR (("Vascular Access Devices"[Mesh]) OR "Central Venous Catheters"[Mesh])) AND ("Randomized Controlled Trial" [Publication Type] OR "Randomized Controlled Trials as Topic"[Mesh]) Filters: Randomized Controlled Trial; Publication date from 2006/01/01 to 2015/12/31; Humans

(("tunneled central venous"[Title/Abstract] OR "tunneled central venous"[Title/Abstract]) OR ("Vascular Access Devices"[Mesh] OR "Central Venous Catheters"[Mesh])) AND ("Randomized Controlled Trial"[Publication Type] OR "Randomized Controlled Trials as Topic"[Mesh]) AND (Randomized Controlled Trial[ptyp] AND ("2006/01/01"[PDAT] : "2015/12/31"[PDAT]) AND "humans"[MeSH Terms])
