# Supplementary material for: Randomized controlled trials in central vascular access devices: A scoping review
Source: PLoS One. 2017 Mar 21;12(3):e0174164. doi: 10.1371/journal.pone.0174164 (PMC5360326; doi:10.1371/journal.pone.0174164)
Supplement: S1 Table — (DOCX) [file pone.0174164.s002.docx]

**S1_Table. Table of included studies**

| **Author** | **Year** | **Title** |
| --- | --- | --- |
| Douard et. al. [170] | 2006 | Prospective, double-blind, randomized trial of equimolar mixture of nitrous oxide/oxygen to prevent pain induced by insertion of venous access ports in cancer patients |
| Fraenkel et. al. [42] | 2006 | A prospective, randomized trial of rifampicin-minocycline-coated and silver-platinum-carbon-impregnated central venous catheters |
| Graf et. al. [109] | 2006 | Sutured securement of peripherally inserted central catheters yields fewer complications in pediatric patients |
| Horne et. al.[168] | 2006 | Use of Heparin versus Lepirudin Flushes to Prevent Withdrawal Occlusion of Central Venous Access Devices |
| Karakitsos  et. al.[64] | 2006 | Real-time ultrasound-guided catheterisation of the internal jugular vein: a prospective comparison with the landmark technique in critical care patients |
| Karthaus et. al. [189] | 2006 | Dalteparin for prevention of catheter-related complications in cancer patients with central venous catheters: final results of a double-blind, placebo-controlled phase III trial |
| Lemyre et. al. [138] | 2006 | How effective is tetracaine 4% gel, before a peripherally inserted central catheter, in reducing procedural pain in infants: a randomized double-blind placebo controlled trial |
| Milling et. al. [84] | 2006 | Randomized controlled trial of single-operator vs. two-operator ultrasound guidance for internal jugular central venous cannulation |
| Moll et. al. [187] | 2006 | Phase II trial of alfimeprase, a novel-acting fibrin degradation agent, for occluded central venous access devices |
| Osma et. al. [78] | 2006 | Efficacy of antiseptic-impregnated catheters on catheter colonization and catheter-related bloodstream infections in patients in an intensive care unit |
| Pawlik et. al. [175] | 2006 | Evaluation of CVC-set |
| Ruud et. al. [184] | 2006 | Low-dose warfarin for the prevention of central line-associated thromboses in children with malignancies—a randomized, controlled study |
| Taddio et. al. [124] | 2006 | Intravenous morphine and topical tetracaine for treatment of pain in preterm neonates undergoing central line placement |
| van den Hoogen et. al. [181] | 2006 | In-line filters in central venous catheters in a neonatal intensive care unit |
| Abdelkefi et. al. [38] | 2007 | Use of heparin-coated central venous lines to prevent catheter-related bloodstream infection |
| Casey et. al. [48] | 2007 | A prospective clinical trial to evaluate the microbial barrier of a needleless connector |
| Esteve et. al. [46] | 2007 | Bloodstream infection related to catheter connections: a prospective trial of two connection systems |
| Ezri et. al. [44] | 2007 | Correct depth of insertion of right internal jugular central venous catheters based on external landmarks: avoiding the right atrium |
| Filipi et. al. [195] | 2007 | Fusidic acid and heparin lock solution for the prevention of catheter-related bloodstream infections in critically ill neonates: a retrospective study and a prospective, randomized trial |
| Fuentes Pumarola et. al.[40] | 2007 | Comparative study of maintenance of patency of triple lumen central venous catheter |
| Gebhard et. al.[39] | 2007 | The accuracy of electrocardiogram-controlled central line placement |
| Hockley et. al.[106] | 2007 | Efficacy of the CathRite system to guide bedside placement of peripherally inserted central venous catheters in critically ill patients: a pilot study |
| Kalfon et. al.[66] | 2007 | Comparison of silver-impregnated with standard multi-lumen central venous catheters in critically ill patients |
| Karakitsos et. al.[63] | 2007 | Ultrasound-guided "low approach" femoral vein catheterization in critical care patients results in high incidence of deep vein thrombosis |
| Mimoz et. al.[82] | 2007 | Chlorhexidine-based antiseptic solution vs alcohol-based povidone-iodine for central venous catheter care |
| Niers et. al.[176] | 2007 | Prevention of catheter-related venous thrombosis with nadroparin in patients receiving chemotherapy for hematologic malignancies: a randomized, placebo-controlled study |
| Shah et. al.[126] | 2007 | A randomized, controlled trial of heparin versus placebo infusion to prolong the usability of peripherally placed percutaneous central venous catheters (PCVCs) in neonates: the HIP (Heparin Infusion for PCVC) study |
| Xiao et. al.[90] | 2007 | Video-based training increases sterile-technique compliance during central venous catheter insertion |
| Bowers et. al.[117] | 2008 | Comparison of occlusion rates by flushing solutions for peripherally inserted central catheters with positive pressure Luer-activated devices |
| Goossens et. al.[154] | 2008 | Functional evaluation of conventional 'Celsite' venous ports versus 'Vortex' ports with a tangential outlet: a prospective randomised pilot study |
| Izquierdo Fuentes et. al.[67] | 2008 | Sterile film as a barrier method in central venous catheter placement |
| Lago et. al.[139] | 2008 | Remifentanil for percutaneous intravenous central catheter placement in preterm infant: a randomized controlled trial |
| Mer et. al.[85] | 2008 | Central Venous Catheterization: A Prospective, Randomized, Double-Blind Study |
| Periard et. al.[130] | 2008 | Randomized controlled trial of peripherally inserted central catheters vs. peripheral catheters for middle duration in-hospital intravenous therapy |
| Sanders et. al.[166] | 2008 | A prospective double-blind randomized trial comparing intraluminal ethanol with heparinized saline for the prevention of catheter-associated bloodstream infection in immunosuppressed haematology patients |
| Teichgraber et. al.[142] | 2009 | A comparison of clinical outcomes with regular- and low-profile totally implanted central venous port systems |
| Valles et. al.[94] | 2008 | Prospective randomized trial of 3 antiseptic solutions for prevention of catheter colonization in an intensive care unit for adult patients |
| van Rooden et. al.[163] | 2008 | Prevention of coagulase-negative staphylococcal central venous catheter-related infection using urokinase rinses: a randomized double-blind controlled trial in patients with hematologic malignancies |
| Agarwal et. al.[36] | 2009 | Ultrasonography: A novel approach to central venous cannulation |
| Alic et. al.[34] | 2009 | Ultrasound-guided catheterization of the subclavian vein: a prospective comparison with the landmark technique in ICU patients |
| Anton et. al.[33] | 2009 | Heparin-bonded central venous catheters do not reduce thrombosis in infants with congenital heart disease: a blinded randomized, controlled trial |
| Biffi et. al.[159] | 2009 | Best choice of central venous insertion site for the prevention of catheter-related complications in adult patients who need cancer therapy: a randomized trial |
| Britt et. al.[52] | 2009 | The impact of central line simulation before the ICU experience |
| Camargo et. al.[50] | 2009 | Double-lumen central venous catheters impregnated with chlorhexidine and silver sulfadiazine to prevent catheter colonisation in the intensive care unit setting: a prospective randomised study |
| Campos et. al.[49] | 2009 | Central venous catheterization: A randomized comparison between external and internal jugular access |
| Cesaro et. al.[172] | 2009 | Prospective, randomized trial of two different modalities of flushing central venous catheters in pediatric patients with cancer |
| De Cicco et. al.[196] | 2009 | Early and short-term acenocumarine or dalteparin for the prevention of central vein catheter-related thrombosis in cancer patients: a randomized controlled study based on serial venographies |
| Garland et. al.[111] | 2009 | Pilot trial to compare tolerance of chlorhexidine gluconate to povidone-iodine antisepsis for central venous catheter placement in neonates |
| Hagau et. al.[71] | 2009 | Central venous catheter colonization and catheter-related bloodstream infections in critically ill patients: a comparison between standard and silver-integrated catheters |
| Islam et. al.[68] | 2009 | Comparative study of stress response to central venous cannulation under local anesthesia and general anesthesia in patients undergoing open heart surgery |
| Johansson et. al.[190] | 2009 | Patients' perceptions of having a central venous catheter or a totally implantable subcutaneous port system-results from a randomised study in acute leukaemia |
| Khalidi et. al.[140] | 2009 | Impact of the positive pressure valve on vascular access device occlusions and bloodstream infections |
| Knebel et. al.[149] | 2009 | Randomized clinical trial of a modified Seldinger technique for open central venous cannulation for implantable access devices |
| Lee et. al.[89] | 2009 | Comparison of the bedside central venous catheter placement techniques: landmark vs electrocardiogram guidance |
| Nocito et. al.[144] | 2009 | Randomized clinical trial comparing venous cutdown with the Seldinger technique for placement of implantable venous access ports |
| Palepu et. al.[76] | 2009 | Impact of ultrasonography on central venous catheter insertion in intensive care |
| Ruschulte et. al.[99] | 2009 | Prevention of central venous catheter related infections with chlorhexidine gluconate impregnated wound dressings: a randomized controlled trial |
| Schweickert et. al.[127] | 2009 | A randomized, controlled trial evaluating postinsertion neck ultrasound in peripherally inserted central catheter procedures |
| Timsit et. al.[95] | 2009 | Chlorhexidine-impregnated sponges and less frequent dressing changes for prevention of catheter-related infections in critically ill adults: A randomized controlled trial |
| Vandoni et. al.[161] | 2009 | Randomised comparison of complications from three different permanent central venous access systems |
| Vokurka et. al.[93] | 2009 | Once- versus twice-weekly changing of central venous catheter occlusive dressing in intensive chemotherapy patients: results of a randomized multicenter study |
| Young et. al.[180] | 2009 | Warfarin thromboprophylaxis in cancer patients with central venous catheters (WARP): an open-label randomised trial |
| Aouad et. al.[31] | 2010 | Femoral vein cannulation performed by residents: a comparison between ultrasound-guided and landmark technique in infants and children undergoing cardiac surgery |
| Birch et. al.[118] | 2010 | A randomised, controlled trial of heparin in total parenteral nutrition to prevent sepsis associated with neonatal long lines: the Heparin in Long Line Total Parenteral Nutrition (HILLTOP) trial |
| Bisseling et. al.[200] | 2010 | Taurolidine lock is highly effective in preventing catheter-related bloodstream infections in patients on home parenteral nutrition: a heparin-controlled prospective trial |
| Bracho-Blanchet et. al.[24] | 2010 | Usefulness of intravenous heparin to prevent thrombosis of central venous catheter in children |
| Dettenkofer et. al.[47] | 2010 | Skin disinfection with octenidine dihydrochloride for central venous catheter site care: a double-blind, randomized, controlled trial |
| Evans et. al.[45] | 2010 | Simulation training in central venous catheter insertion: improved performance in clinical practice |
| Feng et. al.[112] | 2010 | Comparison of the outcomes of central venous catheters inserted from the left side and right side: A prospective randomized controlled study. [Chinese] |
| Gabrail et. al.[194] | 2010 | TROPICS 1: a phase III, randomized, double-blind, placebo-controlled study of tenecteplase for restoration of function in dysfunctional central venous catheters |
| Hill et. al.[107] | 2010 | A silver-alginate-coated dressing to reduce peripherally inserted central catheter (PICC) infections in NICU patients: a pilot randomized controlled trial |
| Ishikawa et. al.[70] | 2010 | Maximal sterile barrier precautions do not reduce catheter-related bloodstream infections in general surgery units: a multi-institutional randomized controlled trial |
| Jain et. al.[191] | 2010 | A randomised trial comparing two types of chronic indwelling central venous catheter (CVC) devices used for the delivery of chemotherapy to patients with non-haematological malignancy-peripherally inserted central venous catheters (PICC) vs subcutaneous |
| Lee et. al.[54] | 2010 | Pre-measured length using landmarks on posteroanterior chest radiographs for placement of the tip of a central venous catheter in the superior vena cava |
| Matzie et. al.[86] | 2010 | Comparison of web-based and classroom-based training programs for point-of care, real-time ultrasound-guided central venous catheter placement |
| Michel et. al.[134] | 2010 | Sevoflurane for central catheter placement in neonatal intensive care: a randomized trial |
| Mitre et. al.[81] | 2010 | Ultrasound-guided external jugular vein cannulation for central venous access by inexperienced trainees |
| Ong et. al. [132] | 2010 | Prospective randomized comparative evaluation of proximal valve polyurethane and distal valve silicone peripherally inserted central catheters |
| Ovezov et. al. [77] | 2010 | Effectiveness and safety of the internal jugular vein catheterization in pediatrics: Ultrasound navigation vs anatomical landmarks (A prospective, randomized, double-blind study) |
| Schroeder et. al. [183] | 2010 | A continuous heparin infusion does not prevent catheter-related thrombosis in infants after cardiac surgery* |
| Slobbe et. al. [164] | 2010 | Prevention of catheter-related bacteremia with a daily ethanol lock in patients with tunnelled catheters: a randomized, placebo-controlled trial |
| Smith et. al. [96] | 2010 | Simulation training and its effect on long-term resident performance in central venous catheterization |
| Uslu et. al. [123] | 2010 | The effect of low-dose heparin on maintaining peripherally inserted percutaneous central venous catheters in neonates |
| Walz et. al. [92] | 2010 | Anti-infective external coating of central venous catheters: A randomized, noninferiority trial comparing 5-fluorouracil with chlorhexidine/silver sulfadiazine in preventing catheter colonization |
| Andreatta et. al. [119] | 2011 | Simulation-based training improves applied clinical placement of ultrasound-guided PICCs |
| Biffi et. al. [160] | 2011 | No impact of central venous insertion site on oncology patients' quality of life and psychological distress. A randomized three-arm trial |
| Burlacu et. al. [198] | 2011 | Remifentanil for the insertion and removal of long-term central venous access during monitored anesthesia care |
| Di Carlo et. al. [157] | 2011 | Could antibiotic prophylaxis be not necessary to implant totally implantable venous access devices? Randomized prospective study |
| Ferreira Chacon et. al. [156] | 2011 | Randomized study of minocycline and edetic acid as a locking solution for central line (port-a-cath) in children with cancer |
| Fragou et. al. [41] | 2011 | Real-time ultrasound-guided subclavian vein cannulation versus the landmark method in critical care patients: a prospective randomized study |
| Hedén et. al. [152] | 2011 | Effect of morphine in needle procedures in children with cancer |
| Hemels et. al. [108] | 2011 | Prevention of neonatal late-onset sepsis associated with the removal of percutaneously inserted central venous catheters in preterm infants |
| Ishizuka et. al. [69] | 2011 | Dressing change reduces the central venous catheter-related bloodstream infection |
| Jun et. al. [102] | 2011 | Time limit research on guide wire retrieve in PICC insertion. |
| Kang et. al. [65] | 2011 | Influence of shoulder position on central venous catheter tip location during infraclavicular subclavian approach |
| Karanlik et. al. [150] | 2011 | The role of antibiotic prophylaxis in totally implantable venous access device placement: results of a single-center prospective randomized trial |
| Karpanen et. al. [179] | 2011 | Do silver-coated needleless intravascular catheter connectors reduce microbial contamination? |
| Kaye et. al. [62] | 2011 | The importance of training for ultrasound guidance in central vein catheterization |
| Khouli et. al. [61] | 2011 | Performance of medical residents in sterile techniques during central vein catheterization: randomized trial of efficacy of simulation-based training |
| Knebel et. al. [148] | 2011 | Insertion of totally implantable venous access devices: an expertise-based, randomized, controlled trial (NCT00600444) |
| Kocum et. al. [60] | 2011 | An alternative central venous route for cardiac surgery: supraclavicular subclavian vein catheterization |
| Krikava et. al. [59] | 2011 | Polyhexanide anti-infective coating of central venous catheters in prevention of catheter colonization and bloodstream infection: Study HC-G-H-0507 |
| Marcatto et. al. [135] | 2011 | EMLA versus glucose for PICC insertion: a randomised triple-masked controlled study |
| Pedrolo et. al.[75] | 2011 | Clinical controlled trial on central venous catheter dressings |
| Perez-Parra et. al. [185] | 2011 | Prospective, randomised study of selective versus routine culture of vascular catheter tips: Patient outcome, antibiotic use and laboratory workload |
| Wang et. al.[122] | 2011 | Influence of self-management education on patients with peripherally inserted central catheters |
| Alport et. al.[120] | 2012 | Bard PowerPICC Solo2 vs Cook Turbo-Ject: A Tale of Two PICCs |
| Antonelli et. al. [32] | 2012 | Comparison of triple-lumen central venous catheters impregnated with silver nanoparticles (AgTive®) vs conventional catheters in intensive care unit patients |
| Apiliogullari et. al. [30] | 2012 | Is a neutral head position as effective as head rotation during landmark-guided internal jugular vein cannulation? Results of a randomized controlled clinical trial |
| Arvaniti et. al.[29] | 2012 | Comparison of Oligon catheters and chlorhexidine-impregnated sponges with standard multilumen central venous catheters for prevention of associated colonization and infections in intensive care unit patients: a multicenter, randomized, controlled study |
| Atahan et. al. [28] | 2012 | The effect of antiseptic solution in central venous catheter care |
| Ball et. al.[26] | 2012 | Randomized, prospective, observational simulation study comparing residents' needle-guided vs free-hand ultrasound techniques for central venous catheter access |
| Carvalho et. al. [114] | 2012 | Placement of peripherally inserted central catheters in children guided by ultrasound: a prospective randomized, and controlled trial |
| Casey et. al. [197] | 2012 | Microbiological comparison of a silver-coated and a non-coated needleless intravascular connector in clinical use |
| Dümichen et. al.[169] | 2012 | Randomized controlled trial of taurolidine citrate versus heparin as catheter lock solution in paediatric patients with haematological malignancies |
| Handrup et. al. [193] | 2012 | Biofilm formation in long‐term central venous catheters in children with cancer: a randomized controlled open‐labelled trial of taurolidine versus heparin |
| Hitz et. al. [151] | 2012 | Athrombogenic coating of long-term venous catheter for cancer patients: a prospective, randomised, double-blind trial |
| Johnston et. al. [103] | 2012 | The effect of peripherally inserted central catheter (PICC) valve technology on catheter occlusion rates--the 'ELeCTRiC' study |
| Kwakman et. al. [58] | 2012 | Medical-grade honey does not reduce skin colonization at central venous catheter-insertion sites of critically ill patients: a randomized controlled trial |
| Lamkinsi et. al. [56] | 2012 | [Internal jugular venous cannulation: what is the best approach?] |
| Latif et. al. [55] | 2012 | Teaching aseptic technique for central venous access under ultrasound guidance: a randomized trial comparing didactic training alone to didactic plus simulation-based training |
| Lim et. al. [88] | 2012 | Effect of the bevel direction of puncture needle on success rate and complications during internal jugular vein catheterization* |
| Marmol et. al. [178] | 2012 | Central catheter dressing in a simulator: the effects of tutor's assistance or self-learning tutorial |
| Marsteller et. al. [177] | 2012 | A multicenter, phased, cluster-randomized controlled trial to reduce central line-associated bloodstream infections in intensive care units* |
| Miyagaki et. al. [133] | 2012 | Performance comparison of peripherally inserted central venous catheters in gastrointestinal surgery: a randomized controlled trial |
| Phipps et. al. [129] | 2012 | A randomized trial of the Vein Viewer versus standard technique for placement of peripherally inserted central catheters (PICCs) in neonates |
| Poletti et. al. [73] | 2012 | Central venous catheter in the short term and local infections: surgical anchor versus 2-octylcyano acrylate |
| Pontes-Arruda et. al. [72] | 2012 | Influence of parenteral nutrition delivery system on the development of bloodstream infections in critically ill patients: an international, multicenter, prospective, open-label, controlled study--EPICOS study |
| Schallom et. al. [97] | 2012 | Heparin or 0.9% sodium chloride to maintain central venous catheter patency: a randomized trial |
| Timsit et. al. [182] | 2012 | Randomized controlled trial of chlorhexidine dressing and highly adhesive dressing for preventing catheter-related infections in critically ill adults |
| Alba et. al. [35] | 2013 | Faculty staff-guided versus self-guided ultrasound training for internal medicine residents |
| Aziz et. al. [27] | 2013 | Subclavian vein catheterization: Supraclavicular versus infraclavicular approach |
| Barreras et. al. [201] | 2013 | Clinical efficacy and safety of Securflux®, an anti-reflux device for intravenous infusion |
| Bowers et. al. [199] | 2013 | Using a multimedia presentation to improve patient understanding and satisfaction with informed consent for minimally invasive vascular procedures: A pilot study |
| Bruzoni et. al. [173] | 2013 | A prospective randomized trial of ultrasound- vs landmark-guided central venous access in the pediatric population |
| Byon et. al. [51] | 2013 | Comparison between ultrasound-guided supraclavicular and infraclavicular approaches for subclavian venous catheterization in children--a randomized trial |
| Fan et. al. [113] | 2013 | Randomized comparison of cancer chemotherapy patient's degree of comfort of two methods of peripherally inserted central venous catheters placement. |
| Fenik et. al. [43] | 2013 | Prepackaged central line kits reduce procedural mistakes during central line insertion: a randomized controlled prospective trial |
| Goossens et. al. [155] | 2013 | Comparing normal saline versus diluted heparin to lock non-valved totally implantable venous access devices in cancer patients: a randomised, non-inferiority, open trial |
| Handrup et. al. [192] | 2013 | Central venous catheters and catheter locks in children with cancer: a prospective randomized trial of taurolidine versus heparin |
| Katheria et. al. [141] | 2013 | A randomized controlled trial of ultrasound-guided peripherally inserted central catheters compared with standard radiograph in neonates |
| Konstantinou et. al. [188] | 2013 | Central vascular catheters versus peripherally inserted central catheters in nurse anesthesia. A perspective within the Greek health system |
| Lavau-Denes et. al. [147] | 2013 | Prophylaxis of catheter-related deep vein thrombosis in cancer patients with low-dose warfarin, low molecular weight heparin, or control: a randomized, controlled, phase III study |
| Rosen et. al. [143] | 2013 | Massage for perioperative pain and anxiety in placement of vascular access devices |
| Abualenain et. al. [37] | 2014 | Comparing standard versus video-based teaching for ultrasound-guided internal jugular central venous catheter access for fourth-year medical students |
| Biffi et. al. [158] | 2014 | Cost effectiveness of different central venous approaches for port placement and use in adult oncology patients: evidence from a randomized three-arm trial |
| Broadhurst et. al. [116] | 2014 | PICC Catheter Securement: A Randomized Controlled Trial in the Home Care Setting |
| Caparas et. al. [115] | 2014 | Does a novel method of PICC insertion improve safety? |
| Decembrino et. al. [171] | 2014 | Lock-therapy with ethanol for the salvage of colonized long-term central venous catheter: Experience in oncohematological pediatric patients |
| Glauser et. al. [110] | 2014 | BED-Side Versus Fluoroscopically Guided Insertion of PICCS: Prospective Randomized Trial |
| Hedén et. al. [153] | 2014 | Effect of high-dose paracetamol on needle procedures in children with cancer--an RCT |
| Hosseini et. al.[105] | 2014 | Early outcome of peripherally inserted central catheter versus peripheral IV line in very low birth weight neonates |
| Itkin et. al. [104] | 2014 | Peripherally inserted central catheter thrombosis - Reverse tapered versus nontapered catheters: A randomized controlled study |
| Li et. al. [137] | 2014 | A randomised, controlled trial comparing the long-term effects of peripherally inserted central catheter placement in chemotherapy patients using B-mode ultrasound with modified Seldinger technique versus blind puncture |
| Lyons et. al. [136] | 2014 | A randomized controlled comparison of flushing protocols in home care patients with peripherally inserted central catheters |
| Marcy et. al. [146] | 2014 | Prospective Randomized Trial Comparing Distal (Arm Port) to Central (Chest Port) Technique in Ambulatory Cancer Patients |
| Miao et. al. [145] | 2014 | Randomized clinical trial comparing ultrasound-guided procedure with the Seldinger's technique for placement of implantable venous ports |
| Oh et. al. [79] | 2014 | The influence of the direction of J-tip on the placement of a subclavian catheter: Real time ultrasound-guided cannulation versus landmark method, a randomized controlled trial |
| Panagiotounakou et. al. [131] | 2014 | Peripherally inserted central venous catheters: frequency of complications in premature newborn depends on the insertion site |
| Patel et. al. [186] | 2014 | Comparison of peripherally inserted central venous catheters (PICC) versus subcutaneously implanted port-chamber catheters by complication and cost for patients receiving chemotherapy for non-haematological malignancies |
| Pedrolo et. al. [101] | 2014 | Chlorhexidine and gauze and tape dressings for central venous catheters: a randomized clinical trial |
| Pedrolo et. al. [74] | 2014 | Chlorhexidine-impregnated dressing for central venous catheter: pilot clinical trial |
| Pittiruti et. al. [128] | 2014 | A prospective, randomized comparison of three different types of valved and non-valved peripherally inserted central catheters |
| Rando et. al. [100] | 2014 | Ultrasound-guided internal jugular vein catheterization: a randomized controlled trial |
| Samantaray et. al. [98] | 2014 | Effects of fentanyl on procedural pain and discomfort associated with central venous catheter insertion: A prospective, randomized, double-blind, placebo controlled trial |
| Shin et. al. [125] | 2014 | A comparative study of two remifentanil doses for procedural pain in ventilated preterm infants: a randomized, controlled study |
| Wang et. al. [91] | 2014 | A prospective randomized controlled trial on effect of norvancomycin tube sealing for prevention of central venous catheter-related infection in critical patients |
| Worth et. al. [162] | 2014 | Ethanol versus heparin locks for the prevention of central venous catheter-associated bloodstream infections: a randomized trial in adult haematology patients with Hickman devices |
| Xia et. al. [121] | 2014 | Role of modified Seldinger technique combined with vascular ultrasonography in the placement of peripherally inserted central catheters in patients with breast cancer undergoing postoperative chemotherapy |
| Yamamoto et. al. [174] | 2014 | Efficacy of 1.0% chlorhexidine-gluconate ethanol compared with 10% povidone-iodine for long-term central venous catheter care in hematology departments: A prospective study |
| Boersma et. al. [25] | 2015 | Concentrated citrate locking in order to reduce the long-term complications of central venous catheters: a randomized controlled trial in patients with hematological malignancies |
| Klek et. al. [167] | 2015 | Taurolidine lock in home parenteral nutrition in adults: results from an open-label randomized controlled clinical trial |
| Laiq et. al. [57] | 2015 | Central venous catheterization and cardiac surgeries |
| Lee et. al. [53] | 2015 | Comparison of Needle Insertion and Guidewire Placement Techniques During Internal Jugular Vein Catheterization: The Thin-Wall Introducer Needle Technique Versus the Cannula-Over-Needle Technique |
| Maecken et. al.[87] | 2015 | Ultrasound-guided catheterisation of the subclavian vein: freehand vs needle-guided technique |
| Mimoz et. al.[83] | 2015 | Skin antisepsis with chlorhexidine-alcohol versus povidone iodine-alcohol, with and without skin scrubbing, for prevention of intravascular-catheter-related infection (CLEAN): an open-label, multicentre, randomised, controlled, two-by-two factorial trial |
| Muller et. al.[80] | 2015 | Transfusion of fresh-frozen plasma in critically ill patients with a coagulopathy before invasive procedures: a randomized clinical trial (CME) |
| Schoot et. al.[165] | 2015 | Prevention of central venous catheter-associated bloodstream infections in paediatric oncology patients using 70% ethanol locks: A randomised controlled multi-centre trial |
