# Supplementary material for: Randomized controlled trials in central vascular access devices: A scoping review
Source: PLoS One. 2017 Mar 21;12(3):e0174164. doi: 10.1371/journal.pone.0174164 (PMC5360326; doi:10.1371/journal.pone.0174164)
Supplement: S2 Table — (DOCX) [file pone.0174164.s003.docx]

**S2 Table: Table 3: Study themes by categories (N = 178 RCTs)**

| **Infection control** | **N** | **Insertion** | **N** | **Education** | **N** | **Catheter and added materials** | **N** | **Dressing and securement** | **N** | **Patency** | **N** |
| --- | --- | --- | --- | --- | --- | --- | --- | --- | --- | --- | --- |
| Antibiotic prophylaxis | 2 | Insertion: pain | 13 | Education: insertion | 5 | Catheter type | 8 | Dressing | 11 | Flush | 5 |
| Barrier precaution pre insertion | 2 | Insertion: technique | 51 | Education: simulation | 7 | Catheter material | 18 | Securement | 1 | Lock solution | 22 |
| Bundle intervention | 1 | Insertion: other | 2 | Patient education | 2 | Filter | 1 |  |  | Anticoagulant prophylaxis | 6 |
| Other: catheter tip culture method | 1 | Post insertion CVAD position check | 1 |  |  | Administration sets | 1 |  |  | Unblocking solution | 2 |
| Removal technique: infection | 1 |  |  |  |  | Connectors | 7 |  |  |  |  |
| Skin prep/ decontamination | 8 |  |  |  |  |  |  |  |  |  |  |
| **Total** | 15 |  | 67 |  | 14 |  | 35 |  | 12 |  | 35 |
