# Supplementary material for: Randomized controlled trials in central vascular access devices: A scoping review
Source: PLoS One. 2017 Mar 21;12(3):e0174164. doi: 10.1371/journal.pone.0174164 (PMC5360326; doi:10.1371/journal.pone.0174164)
Supplement: S3 Table — *One catheter material study had also a theme of heparin flush. One removal technique study included insertion technique, but this is only included in one category. RCT: Randomized Controlled Trial; CVAD: Central Venous Access Device; NTCVAD: Non-tunneled Central Venous Access Device; PICC: Peripherally Inserted Central Catheter; TIVAD: Totally Implantable Vascular Access Device; CVAD NS: Central Venous Access Device Not Specified; PICU: Pediatric Intensive Care Unit; NICU: Neonatal Intensive Care Unit. (DOCX) [file pone.0174164.s004.docx]

**S3 Table: Table 4: Study themes by CVAD type (N = 178 studies)­­**

| **Theme** | **NTCVAD** | **PICC** | **TIVAD** | **Tunneled** | **Combined** | **CVAD NS** | **Total** |
| --- | --- | --- | --- | --- | --- | --- | --- |
| **Infection control** |  |  |  |  |  |  |  |
| Antibiotic prophylaxis | 0 | 0 | 2 | 0 | 0 | 0 | 2 |
| Barrier precaution pre insertion | 2 | 0 | 0 | 0 | 0 | 0 | 2 |
| Bundle intervention | 0 | 0 | 0 | 0 | 0 | 1 | 1 |
| Catheter tip culture: method | 0 | 0 | 0 | 0 | 1 | 0 | 1 |
| Removal technique: infection | 0 | 1 | 0 | 0 | 0 | 0 | 1 |
| Skin prep/decontamination | 6 | 1 | 0 | 0 | 0 | 1 | 8 |
| **Insertion-related** |  |  |  |  |  |  |  |
| Insertion: pain | 2 | 6 | 3 | 1 | 1 | 0 | 13 |
| Insertion: technique | 28 | 12 | 8 | 1 | 1 | 1 | 51 |
| Insertion: other | 2 | 0 | 0 | 0 | 0 | 0 | 2 |
| Post-insertion CVAD position check | 0 | 1 | 0 | 0 | 0 | 0 | 1 |
| **Education** |  |  |  |  |  |  |  |
| Education: insertion | 4 | 0 | 0 | 0 | 0 | 1 | 5 |
| Education: simulation | 6 | 1 | 0 | 0 | 0 | 0 | 7 |
| Patient education | 0 | 1 | 0 | 0 | 1 | 0 | 2 |
| **Catheter and added materials** |  |  |  |  |  |  |  |
| Catheter type (CVAD type) | 0 | 2 | 3 | 0 | 3 | 0 | 8 |
| Catheter material (coated catheters/different brands) | 12 | 5 | 1 | 0 | 0 | 0 | 18 |
| Filter | 0 | 0 | 0 | 0 | 1 | 0 | 1 |
| Administration sets | 1 | 0 | 0 | 0 | 0 | 0 | 1 |
| Connectors | 2 | 2 | 0 | 0 | 2 | 1 | 7 |
| **Dressing and securement** |  |  |  |  |  |  |  |
| Dressing | 7 | 3 | 0 | 0 | 1 | 0 | 11 |
| Securement | 1 | 0 | 0 | 0 | 0 | 0 | 1 |
| **Patency** |  |  |  |  |  |  |  |
| Flush | 2 | 1 | 0 | 2 | 0 | 0 | 5 |
| Lock solution | 3 | 4 | 2 | 8 | 5 | 0 | 22 |
| Anticoagulant prophylaxis | 0 | 0 | 1 | 0 | 4 | 1 | 6 |
| Unblocking solution | 0 | 0 | 0 | 0 | 2 | 0 | 2 |
| **Total** | 78 | 40 | 20 | 12 | 22 | 6 | 178 |

*One catheter material study had also a theme of heparin flush. One removal technique study included insertion technique, but this is only included in one category.

RCT: Randomized Controlled Trial; CVAD: Central Venous Access Device; NTCVAD: Non-tunneled Central Venous Access Device; PICC: Peripherally Inserted Central Catheter; TIVAD: Totally Implantable Vascular Access Device; CVAD NS: Central Venous Access Device Not Specified; PICU: Pediatric Intensive Care Unit; NICU: Neonatal Intensive Care Unit
