# Supplementary material for: Randomized controlled trials in central vascular access devices: A scoping review
Source: PLoS One. 2017 Mar 21;12(3):e0174164. doi: 10.1371/journal.pone.0174164 (PMC5360326; doi:10.1371/journal.pone.0174164)
Supplement: S4 Table — RCT: Randomized Controlled Trial; CVAD: Central Venous Access Device; NTCVAD: Non-tunneled Central Venous Access Device; PICC: Peripherally Inserted Central Catheter; TIVAD: Totally Implantable Vascular Access Device; CVAD NS: Central Venous Access Device Not Specified; PICU: Pediatric Intensive Care Unit; NICU: Neonatal Intensive Care Unit; CDC: Centers of Disease Control; MRI: Magnetic Resonance Imaging; USS: Ultra sound sonography. (DOCX) [file pone.0174164.s005.docx]

**S4_Table 5: Study outcomes by CVAD type (433 outcomes)**

|  | **NTCVAD** | **PICC** | **TIVAD** | **Tunneled** | **Combined** | **CVAD NS** | **Total** |
| --- | --- | --- | --- | --- | --- | --- | --- |
| **Patient outcomes** |  |  |  |  |  |  |  |
| Pain | 1 | 6 | 4 | 1 | 1 | 0 | 13 |
| Patient satisfaction | 0 | 3 | 0 | 0 | 2 | 0 | 5 |
| Mortality rate | 2 | 1 | 0 | 0 | 0 | 0 | 3 |
| Psychological distress | 0 | 0 | 1 | 0 | 0 | 0 | 1 |
| Quality of life | 0 | 0 | 1 | 0 | 0 | 0 | 1 |
| Sedation | 0 | 0 | 0 | 0 | 1 | 0 | 1 |
| Vital signs | 1 | 2 | 0 | 0 | 0 | 0 | 3 |
| Self-management ability | 0 | 1 | 0 | 0 | 0 | 0 | 1 |
| Patient comprehension | 0 | 0 | 0 | 0 | 1 | 0 | 1 |
| **Catheter insertion outcomes** |  |  |  |  |  |  |  |
| Successful placement measures | 28 | 12 | 5 | 1 | 1 | 0 | 47 |
| Insertion related complications | 25 | 3 | 9 | 1 | 1 | 0 | 39 |
| Insertion success: performance scores | 9 | 1 | 0 | 0 | 1 | 2 | 13 |
| Requirement of positioning | 1 | 0 | 0 | 0 | 0 | 0 | 1 |
| Utilization of USS | 1 | 0 | 0 | 0 | 0 | 0 | 1 |
| **Catheter complications** |  |  |  |  |  |  |  |
| Occlusion | 2 | 14 | 2 | 3 | 2 | 0 | 23 |
| Patency | 1 | 0 | 1 | 0 | 3 | 0 | 5 |
| Number of thrombolytic/fibrinolysis injections | 0 | 2 | 1 | 0 | 1 | 0 | 4 |
| Anticoagulant treatment | 1 | 0 | 0 | 0 | 0 | 0 | 1 |
| Thrombosis - diagnosed and screened by USS/venography | 2 | 1 | 1 | 0 | 4 | 0 | 8 |
| Thrombosis - not diagnosed by USS/MRI/CT/venography | 2 | 6 | 3 | 2 | 1 | 0 | 14 |
| Thrombosis - diagnosed by USS/MRI/CT/venography | 1 | 2 | 1 | 1 | 1 | 1 | 7 |
| Early removal | 0 | 3 | 3 | 3 | 5 | 0 | 14 |
| Dwell time | 2 | 7 | 0 | 1 | 2 | 0 | 12 |
| Mechanical failure (migration/catheter defects/ malfunction/ infiltration/skin fixation failure) | 2 | 3 | 3 | 0 | 0 | 0 | 8 |
| Complication rate (not clarified) | 1 | 0 | 1 | 3 | 0 | 0 | 5 |
| Catheter dislocation | 0 | 2 | 1 | 0 | 1 | 0 | 4 |
| Catheter fracture | 0 | 2 | 0 | 0 | 0 | 0 | 2 |
| Local edema/ inflammation | 0 | 1 | 0 | 0 | 0 | 0 | 1 |
| Mechanical failure (Other) | 0 | 1 | 0 | 0 | 0 | 0 | 1 |
| **Infective outcomes** |  |  |  |  |  |  |  |
| CRBSI defined by CDC | 15 | 4 | 0 | 4 | 1 | 2 | 26 |
| CRBSI defined by other reference | 9 | 0 | 2 | 3 | 6 | 1 | 21 |
| Catheter-related infection - not defined | 4 | 12 | 5 | 1 | 3 | 0 | 25 |
| Catheter related infection - specified | 2 | 0 | 0 | 0 | 1 | 1 | 4 |
| Catheter related sepsis | 2 | 2 | 2 | 2 | 0 | 0 | 8 |
| CVAD tip colonization | 19 | 1 | 0 | 3 | 4 | 2 | 29 |
| Contamination/ colonization of non-catheter materials including skin and hub | 9 | 1 | 1 | 3 | 4 | 2 | 20 |
| Systemic infection/sepsis | 2 | 2 | 2 | 1 | 1 | 0 | 8 |
| Phlebitis | 0 | 8 | 0 | 0 | 1 | 0 | 9 |
| Local infection/exit site infection | 2 | 0 | 3 | 2 | 1 | 0 | 8 |
| Temperature | 1 | 0 | 1 | 0 | 0 | 0 | 2 |
| Biofilm | 0 | 0 | 0 | 0 | 1 | 0 | 1 |
| **Intervention-related** |  |  |  |  |  |  |  |
| Side effects/tolerability | 6 | 4 | 0 | 3 | 3 | 0 | 16 |
| Bleeding | 1 | 1 | 0 | 1 | 0 | 1 | 4 |
| Skin necrosis | 0 | 0 | 1 | 0 | 0 | 0 | 1 |
| **Health service related** |  |  |  |  |  |  |  |
| Health economy/ cost | 1 | 3 | 2 | 1 | 3 | 1 | 11 |
| Microbiology workload | 0 | 0 | 0 | 0 | 1 | 0 | 1 |
| **Total** | 155 | 111 | 56 | 40 | 58 | 13 | 433 |

RCT: Randomized Controlled Trial; CVAD: Central Venous Access Device; NTCVAD: Non-tunneled Central Venous Access Device; PICC: Peripherally Inserted Central Catheter; TIVAD: Totally Implantable Vascular Access Device; CVAD NS: Central Venous Access Device Not Specified; PICU: Pediatric Intensive Care Unit; NICU: Neonatal Intensive Care Unit; CDC: Centers of Disease Control; MRI: Magnetic Resonance Imaging; USS: Ultra sound sonography
